# Reconnection with nature through empathy: rewiring people and animals by assessing zoo visitors' connection to species and the need for their conservation

**DOI:** 10.3389/fpsyg.2025.1517430

**Published:** 2025-03-17

**Authors:** Raquel Costa, Shenwen Xu, Angela Brandão, Misato Hayashi

**Affiliations:** ^1^Research Department, Japan Monkey Centre, Inuyama, Japan; ^2^Primate Cognition Research Group, Lisbon, Portugal; ^3^Movimento Mulheres pela Primatologia, Florianopolis, Brazil; ^4^Center for the Evolutionary Origins of Human Behavior, Kyoto University, Inuyama, Japan; ^5^Centro de Filosofia das Ciências da Universidade de Lisboa, Lisbon, Portugal; ^6^Department of Child Development, Faculty of Education, Chubu Gakuin University, Seki, Gifu, Japan

**Keywords:** visitor's connection, animal's connection, educational programs, wildlife conservation, animal welfare, zoos

## 1 Introduction

As humans become more sensitive to global warming, due to the visible effects on the planet and our daily lives, particularly our health (for example the increase of zoonotic diseases exacerbated by deforestation), we have an increasing need to reconnect with the natural world (Greenwell et al., 2023; Johansson et al., 2024). In cities, far from forests and savannas, zoos become the bridge to wild habitats, aiming to encourage proximity and connection between people and animals. Zoos allow their visitors to be close to wild animals, in many cases for the first time, while providing them with basic information related to the ecology, behavior, and conservation of these species. In zoos, friendly human-animal associations may further develop into bonds that positively influence both parties (Thomas-Walters et al., 2023). Moreover, visitors with a better learning experience and deeper connections with the animals have a better understanding of biodiversity conservation (Moss et al., 2017), are more critical of bad captive-wildlife tourist attractions (Sampaio et al., 2021), are more willing to donate to conservation actions (Ballantyne et al., 2007; Howell et al., 2019), and have increased desire to visit the animals in the wild [76% of visitors expressing a desire to see animals in their habitat (Adetola and Akinboboye, 2020)]. Most of these studies have been focused on a few African countries, some European countries, the USA, and Australia, largely overlooking non-western countries (McNally et al., 2024). While there are exceptions, such as the studies by Wu et al. (2017) in China and Musa et al. (2015) in Malaysia, these are relatively few in Asia. People's connection to wildlife, and their beliefs and attitudes toward conservation needs, depend on cultural and socio-demographic traits (Ballantyne et al., 2021; Breuer et al., 2018; Thomas-Walters et al., 2023), we currently lack, the understanding of how zoos in some parts of the world can effectively transmit conservation messages tailored to their specific audiences. In Japan, the number of zoo visitors has increased in the last few decades (Davey, 2007) and the physiological effects of zoos were studied. The mandatory quarantine was not imposed on the Japanese population during the pandemic, and many indoor entertainment activities were suspended. Nonetheless, most Japanese zoos did not close their doors during the pandemic and were one of the few tourist attractions available to the public, providing close contact with wildlife. Zoos are considered to positively impact people's mental health through “green prescribing” interventions (Rose and Riley, 2022). Using both psychological (mood states) and physical indicators (i.e., blood pressure, and salivary cortisol and oxytocin levels), Akiyama et al. (2021) found a positive health effect associated with a zoo visit among a sampled population of elderly Japanese. Similarly, Sakagami and Ohta (2010) also found a decrease in blood pressure, increased physical exercise, and improved life sub-scale scores (i.e., generic health-related evaluation questionnaire) in participants of all ages visiting two different Japanese zoos. However, little research has been done on the actual interactions between visitors and zoo animals and their emotional responses and motivation for preserving species. In other words, how connected people are with animals is still poorly understood. Connection sense is defined here as an innate, empathetic bond that visitors may experience toward the animals they observe, ranging from absent to neutral, to a high level of connection. In this Data report, we survey visitants' motivation for conservation support, their feeling of connectedness with the captive animals in the zoo, and to what level they can relate zoonotic transmission between humans and animals. By focusing on a specific species, we provide participants with a precise “target,” which can help them to reflect better and more accurately on the answers. This approach is expected to yield more sincere and insightful responses, as participants are more likely to form genuine connections with particular species. Moreover, targeting specific species allows us to understand which species elicit higher levels of awareness related to the viewing times.

## 2 Methods

### 2.1 Ethical approval

Permission to conduct this study was obtained from the Japan Monkey Centre (No. 2022-05). Consent forms were obtained from all visitors inquired following a short explanation of the study, attesting that responses were anonymous and that the data collected would be used for the study analysis only. No personal data was collected and taking part in the surveys did not interfere with the participant's visit to the zoo. Questionnaires to Chubu Gakuin University students were provided before and after a class on animal welfare and a visit to the Japan Monkey Centre for a behavioral observation in a lecture of Comparative Cognitive Development.

### 2.2 Data sampled

Two zoological parks were included in this study: the Japan Monkey Centre (JMC) and Higashiyama Zoo and Botanical Gardens (HZ), Japan. JMC is home to 60 primate species focusing on this Genus only, while HZ hosts a wide range of animals and plants. Both zoos work closely with researchers and public education. Data were collected from August to November 2022 in JMC and from May to September 2023 in HZ. Participants (aged above 16 years old) were approached from 10:00 to 16:00 during zoo opening hours. Questionnaires were handed out to visitors on paper within the zoos' premises, while non-visitors completed an online version of the questionnaire. University students were provided with a link to the online questionnaire.

### 2.3 Data collection

Participants completed the questionnaire that included demographic information (sex, age, zoo membership), favorite animal, 21 questions related to general aspects of the zoo visit and awareness of wildlife conservation (adapted from Howell et al., 2019 and Skibins and Powell, 2013), and an additional free space for comments. The questions regarding wildlife connection consisted of closed response options and scaling (1 to 7) the level of connection and agreement with items regarding their personal views of wildlife and wildlife conservation (Supplementary Table 1). No definition of connection, wildlife, conservation and welfare was provided to the respondents prior to the questionnaire.

### 2.4 Data analysis and preliminary results

A total of 630 participants completed the questionnaires. Two groups of undergraduate students were given the questionnaire twice: once before and once after visiting the zoo. A total of 56 students replied on both occasions and 53 students replied to the questionnaire either before or after the trip. The individuals who replied twice to the questionnaires were given ID codes to ensure their anonymity and were included in the analysis (others were excluded). These students were divided into 4 groups: (1) S22B stands for the Students of the 2022 Class Before the trip to the zoo, (2) S22A stands for the Students of the 2022 Class After the trip to the zoo, (3) S23B stands for the Students of the 2023 Class Before the trip to the zoo, and (4) S23A stands for the Students of the 2022 Class After the trip to the zoo.

In terms of gender distribution, 43 students identified as female in the university student population, 177 visitors identified as female in HZ, and 159 visitors identified as female in JMC. For male respondents, 11 students identified as male in the students, 123 visitors identified as male in HZ, and 101 visitors identified as male in JMC. Sixteen respondents replied “Other” or did not provide any information regarding their sex.

All undergraduate students belonged to the GenZ generation. We classify Gen Z as individuals born between 1995 and 2015; Millennials between 1980 and 1994; Gen X between 1965 and 1979; and Baby Boomers as those born before 1964 (Supplementary Table 2).

**Figure 1:**
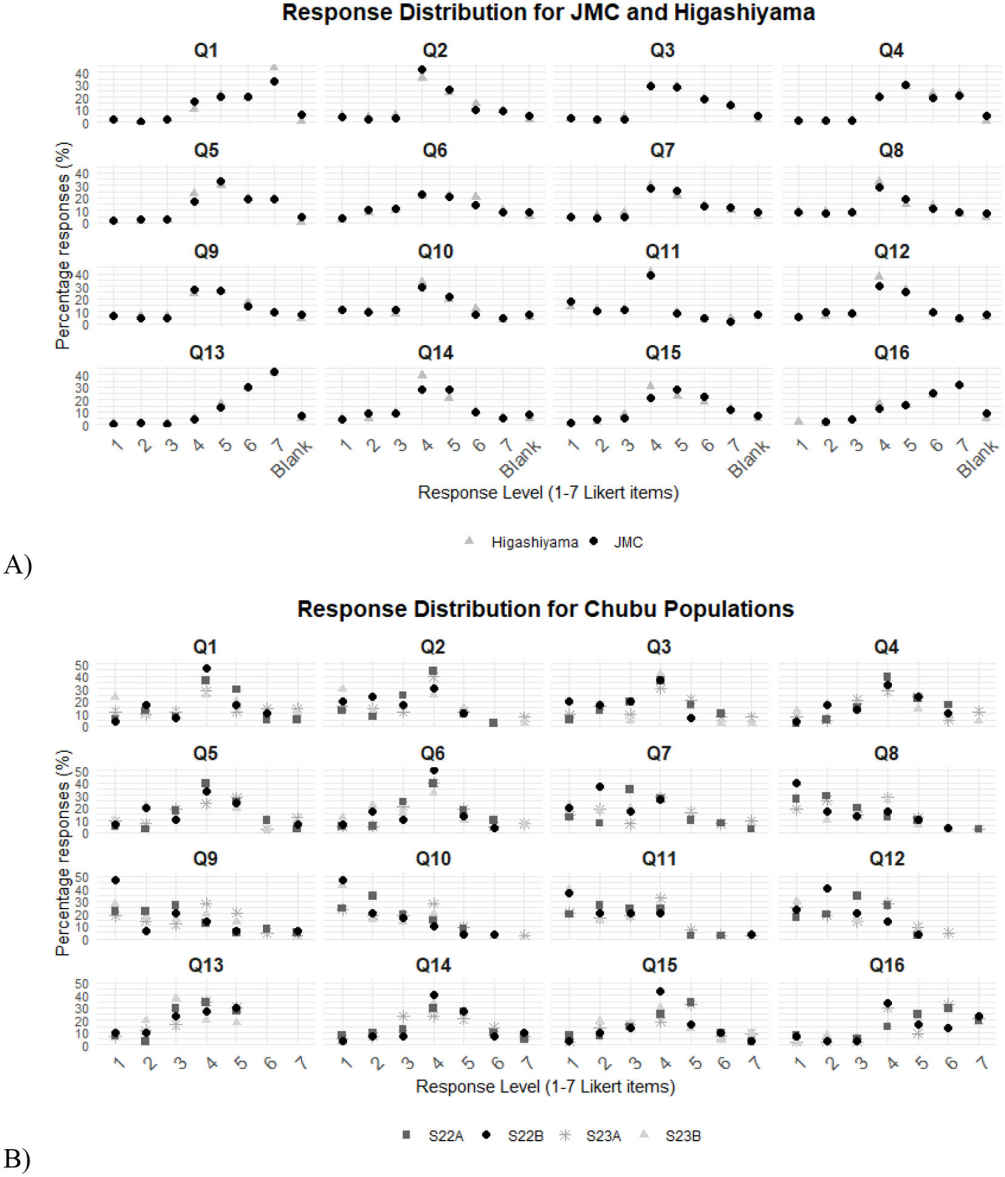
**(A)** Percentage of responses per Likert Scale Item for each Question for the surveyed zoo populations: JMC, Higashiyama Zoo; **(B)** Percentage of responses per Likert Scale Item for each Question for the surveyed students at Chubu Gakuin University. These students were divided into 4 groups: (1) S22B stands for the Students of the 2022 Class Before the trip to the zoo, (2) S22A stands for the Students of the 2022 Class After the trip to the zoo, (3) S23B stands for the Students of the 2023 Class Before the trip to the zoo, and (4) S23A stands for the Students of the 2022 Class After the trip to the zoo.

The percentages of responses related to the rate of connection with the animals reported by participants and the frequency reported for observation and interaction with the animals are presented in Table 1. The closed responses section of Likert Scales are presented in Figure 1. Overall, the HZ population exhibited a more varied response distribution compared to the JMC population. Responses from HZ often showed peaks at different response levels across multiple questions, indicating a higher degree of variability. In contrast, the JMC population demonstrated a more consistent pattern, with responses frequently clustering around specific levels. The Chubu Gakuin student population exhibited a relatively consistent response distribution across the questions.

**Table 1:** Descriptive results of the responses from the sampled populations regarding their favorite animal, the feeling of connection to that animal, and the time spent observing and interacting with that animal.

		**What animal did you form the strongest connection with during your visit? (First three preferences; number of respondents)**															
		**Gorilla**		**Chimpanzee**		**Polar bear**		**Squirrel monkey**		**Monkey**		**Yakushima macaque**					
JMC		79		51				22									
Higashiyama		130		23		19											
Chubu Gakuin	Before	10		12						21							
		After		8		9						16		10					
		1. Please rate the overall connection you felt with the animal.															
		*No connection at all*				*Neutral connection*						*Extremely strong connection*					
		1 (%%)		2 (%%)		3 (%%)		4 (%%)		5 (%%)		6 (%%)		7 (%%)		Blank	
JMC	Regulars	0	0.00%	8	22.86%	6	17.14%	9	25.71%	6	17.14%	2	5.71%	4	11.43%	0	0.00%
	Non-regulars	12	5.53%	24	11.06%	40	18.43%	81	37.33%	28	12.90%	16	7.37%	8	3.69%	8	3.69%
	blank	1	5.56%	1	5.56%	2	11.11%	6	33.33%	4	22.22%	1	5.56%	2	11.11%	1	5.56%
Higashiyama	Regulars	1	2.04%	2	4.08%	6	12.24%	20	40.82%	5	10.20%	9	18.37%	4	8.16%	2	4.08%
	Non-regulars	22	9.69%	21	9.25%	47	20.70%	58	25.55%	42	18.50%	17	7.49%	11	4.85%	9	3.96%
	blank	1	3.57%	3	10.71%	5	17.86%	7	25.00%	5	17.86%	4	14.29%	2	7.14%	1	3.57%
Chubu Gakuin	Before	16	19.75%	10	12.35%	7	8.64%	36	44.44%	9	11.11%	0	0.00%	3	3.70%	0	0.00%
	After	8	9.52%	12	14.29%	15	17.86%	35	41.67%	7	8.33%	2	2.38%	5	5.95%	0	0.00%
		3. How many times did you visit this animal‘s enclosure?									
		1 (%)		2–5 (%)		6–10 (%)		+10 (%)		Blank	
JMC	Regulars	0	0.00%	4	11.43%	6	17.14%	25	71.43%	0	0.00%
	Non-regulars	113	52.07%	80	36.87%	12	5.53%	11	5.07%	1	0.46%
	blank	12	66.67%	4	22.22%	0	0.00%	2	11.11%	0	0.00%
Higashiyama	Regulars	0	0.00%	9	18.37%	3	6.12%	37	75.51%	0	0.00%
	Non-regulars	108	47.58%	79	34.80%	19	8.37%	20	8.81%	1	0.44%
	blank	14	50.00%	9	32.14%	2	7.14%	3	10.71%	0	0.00%
Chubu Gakuin	Before	50	24.27%	126	61.17%	16	7.77%	14	6.80%	0	0.00%
	After	17	20.99%	52	64.20%	4	4.94%	8	9.88%	0	0.00%
		4A. Approximately how long did you spend at this animal's enclosure in total?													
		< 1 min		1–5 min		6–10 min		11–20 min		21–30 min		More than 30 min		Blank	
JMC	Regulars	0	0.00%	6	17.14%	11	31.43%	9	25.71%	1	2.86%	8	22.86%	0	0.00%
	Non-regulars	2	0.92%	74	34.10%	87	40.09%	30	13.82%	10	4.61%	13	5.99%	1	0.46%
	blank	1	5.56%	5	27.78%	7	38.89%	4	22.22%		0.00%	1	5.56%	0	0.00%
Higashiyama	Regulars	1	2.04%	12	24.49%	11	22.45%	2	4.08%	4	8.16%	19	38.78%	0	0.00%
	Non-regulars	2	0.88%	50	22.03%	91	40.09%	33	14.54%	28	12.33%	22	9.69%	1	0.44%
	blank	0	0.00%	5	17.86%	12	42.86%	4	14.29%	3	10.71%	4	14.29%	0	0.00%
		4B. How many times have you visited a zoo, sanctuary, or other habitat to observe your favorite animals?							
		1^st^ time		2∧5 times		6~10 times		More than 10 times	
Chubu Gakuin	Before	34	41.98%	43	53.09%	3	3.70%	1	1.23%
	After	39	46.43%	42	50.00%	3	3.57%	0	0.00%
		5. To what extent did this animal interact with you (i.e. respond to your movements)?									
		It was asleep		It did not interact with me at all		It interacted with me for a few seconds		It interacted with me for a few seconds or more		Blank	
JMC	Regulars	3	8.57%	12	34.29%	11	31.43%	9	25.71%	0	0.00%
	Non-regulars	24	11.06%	61	28.11%	76	35.02%	41	18.89%	15	6.91%
	blank	2	11.11%	1	5.56%	9	50.00%	4	22.22%	2	11.11%
Higashiyama	Regulars	3	6.12%	17	34.69%	14	28.57%	14	28.57%	1	2.04%
	Non-regulars	19	8.37%	81	35.68%	64	28.19%	56	24.67%	5	2.20%
	blank	4	14.29%	8	28.57%	5	17.86%	9	32.14%	2	7.14%
Chubu Gakuin	Before	9	11.11%	27	33.33%	29	35.80%	16	19.75%	0	0.00%
	After	5	5.95%	26	30.95%	33	39.29%	20	23.81%	0	0.00%

The table highlights the three most favored animals for each population, specifically for “regular” (full members of the zoo, who have the annual pass) and “non-regulars” (visitors who do not have full membership of the zoo). Considering the annual pass price, full members are expected to visit the zoo at least 4 times a year. Percentages represent the proportion of respondents of the specific population for each response.

For each population, the analysis of Cronbach's Alpha showed α ≥ 0.9 when combining all 16 questions together. To compare our findings with those of Howell et al. (2019), from which we adapted the questionnaire, we also tested the composite variables using the questions shared between both studies (Supplementary Table 3). The species-specific conservation caring composite variable demonstrated a Cronbach's Alpha similar to that found in Howell et al. (2019), suggesting that our measure of conservation caring is reliable and comparable to that of Howell's study. However, for other composite variables, the internal consistency was less robust. In the cases where there were fewer questions available for analysis, Cronbach's Alpha values ranged from 0.6 to 0.7, indicating questionable internal consistency and suggesting that more items may be needed for these variables to provide a reliable measure.

In addition, focusing on the zoo sampled populations, we conducted a Principal Component Analysis to compare with Howell's findings, in particular for that same composite variable “species-specific conservation caring.” We found a lower correlation (Pearson r-value = 0.3) to that of Howell's study but it still suggests that more individuals care about conservation as stronger their perceived connection to wildlife. Still the difference in the strength of this correlation may be attributed to cultural differences, or sample size.

For preliminary statistical analysis focusing on our data and with the added questions, a Bayesian model was fitted to evaluate the effects of the (1) Connection rates in relation to the level of agreement in Q1–Q16 questions, (2) sex and age classes, (3) duration of animal observation, and (4) interaction with animals. For point 1, the response variable was the items of each question (Q1–Q16) and the predictor variable Connection was divided into “Low” (Likert items 1, 2, and 3) and “High” (items 5, 6, and 7), removing the “4” (medium) responses from the data to address the issue of potential imbalance on a 7-point Likert scale. For points 2 to 4, the response variables were the Connection rate (continuous from 1–7) and the predictor variables were: Gender (women, men); Generation (Baby Boomer, Gen X, Gen Z, and Millennial); Time spent with the animals was categorized between “Short” (< 10 min), “Medium” (between 11 to 30 min) and “High” (more than 30 min); and Interaction made with the animals were categorized between “No” (absent), “Short” (< 5 seconds) or “Long” (more than 5 seconds). We were not able to correlate regular zoo visitors (with more than 10 visits to the zoo) and new visitors as the latter group is much more frequent. The model used a Gaussian family distribution, with 4 chains and 2,000 iterations per chain. All analyses were conducted in R version 4.3.1, with the brms package version 2.21.0. Statistical significance was interpreted through the 95% highest posterior density interval.

For zoo visitors, we found that a stronger connection with favorite animals correlates with a stronger agreement with statements concerning interest in, motivation to protect, and emotional connection to animals. Across the questions Q1–Q15, the estimated effect sizes ranged from small to moderate, with corresponding 95% credible intervals ranging not containing zero. For Q16 (“Getting close to wild animals is dangerous”), the credible interval included zero (E = 0.123, 95% CI = [−0.251, 0.498]), meaning no clear evidence of an association, which suggests that risk assessment of proximity to wildlife does not correlate with the level of connection to the animals (Supplementary Table 4). In addition, we asked participants to choose one or more options regarding (1) their understanding of the consequences of getting close to wild animals in their habitat, and (2) what actions they would take when visiting tourist sites to observe wild animals (Table 2). Noting that each respondent could choose one or more responses, these results indicate that most visitors prioritized the risks of injury and the importance of maintaining a safe distance from wild animals. A high number of visitors also acknowledge the risks of disease and the importance of taking health precautions such as vaccinations and using masks. However, fewer participants prioritize testing for COVID-19 or are willing to pay extra for safer viewing conditions.

**Table 2:** Participants perceptions of consequences of getting close to wild animals and actions participants would take when observing wild animal (number of responses considering that each participant could chose more than one option).

**Getting close to wild animals may cause**		**To view wild animals, I would…**	
Disease	179	Get vaccines	223
Injury	354	Not going while sick	258
Better picture	56	Test covid	79
Clearer observation of the animal	252	Use mask	162
Habituating the animal	106	Pay extra	27

**Figure 2:**
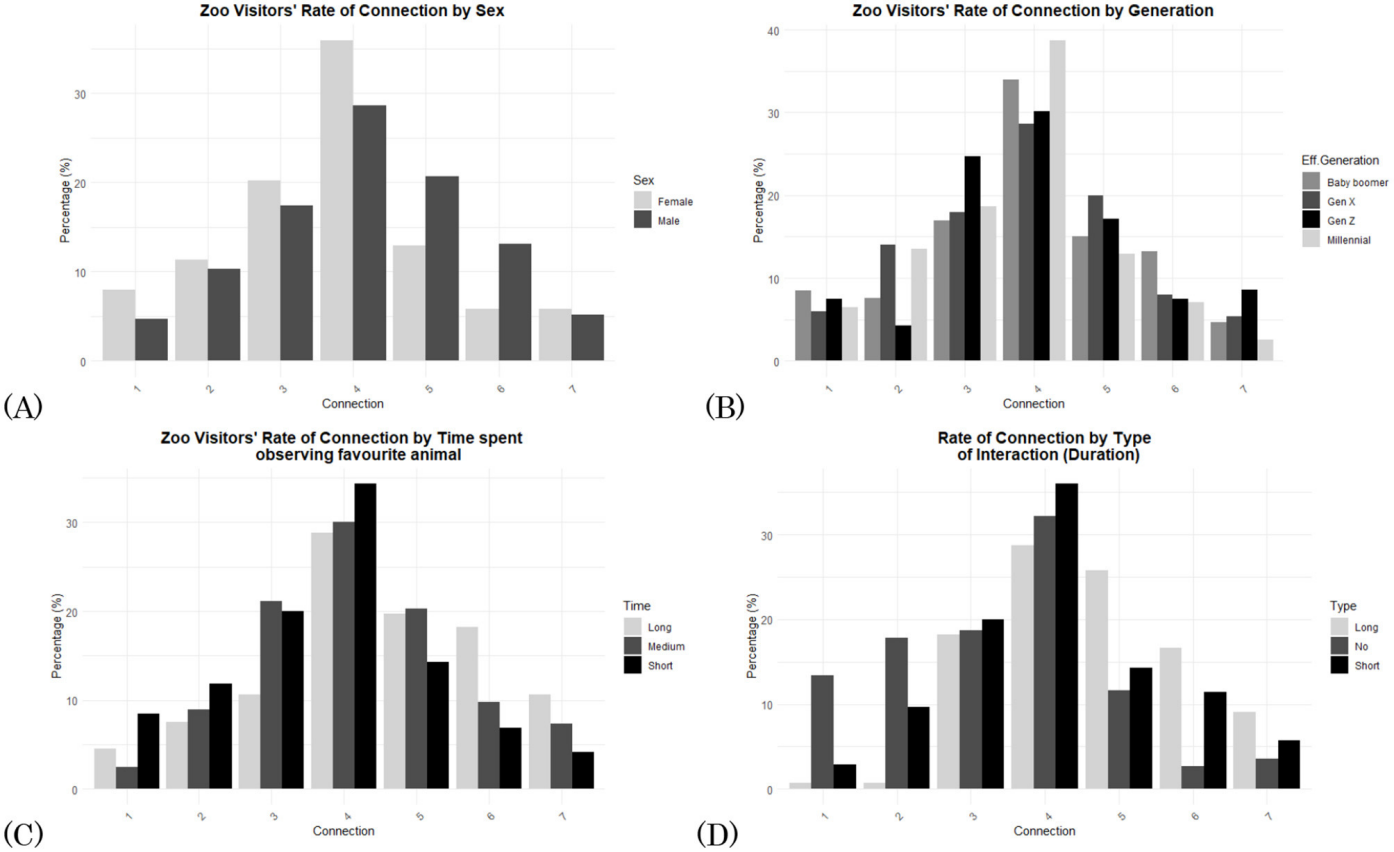
**(A)** Reported connection rates to favorite species among zoo visitors by sex; **(B)** Reported connection rates to favorite species among zoo visitors by generation; **(C)** Reported connection rates to favorite species among zoo visitors, in relation to the time spent observing the favorite animal; **(D)** Reported connection rates among favorite species zoo visitors, in relation to the time spent interacting with their favorite animal.

Regarding sex and age class differences among participants in regards to their connection toward animals, we found sex-related differences in connection to favorite animals with the mean Connection rates for males being 0.35 points higher (95% CI: 0.10 to 0.61) compared to females. However, no significant differences were noted across generations when comparing “Baby boomer” to: Generation X Estimate: –−0.10, 95% CI: −0.48 to 0.27; Generation Z Estimate: 0.04, 95% CI: −0.37 to 0.44, and Millennials Estimate: −0.29, 95% CI: −0.66 to 0.08. Moreover, we found no significant differences in Connection rates related to the duration of animal observation per visit (compared to “Long” time, “Medium” Estimate: −0.02, 95% CI: −2.44 to 2.36; “Short” Estimate: −0.02, 95% CI: −2.46 to 2.42), neither in the levels of interaction with the animals when comparing “Long” interactions with “No” Type Estimate: 0.00, 95% CI: −2.38 to 2.40, and “Short” Type Estimate: −0.04, 95% CI:−2.46 to 2.43 (Figure 2, see also Supplementary Tables 5, 6 for descriptive results).

## 3 Commentary

### 3.1 Proximity to animals and its associated risks

Although we found a positive correlation between the connection rate to the animals and the Likert scale items in each question—indicating that a stronger connection with animals promotes a deeper desire to learn about and protect species (Carr, 2016; Howell et al., 2019)—the participants in this study often disagreed with the risks associated with approaching wild animals. In fact, more people answered that “getting close to wild animals may cause injury” (179 respondents) and “disease transmission” (179) while 252 respondents replied that this would grant them a clearer observation of the animal. This shows that despite being supportive of conservation and educational causes, visitors may still romanticize their connection to animals, underestimating the risk of close encounters with animals given their lack of experience beyond observing animals from the safety of a glass or enclosure's grid. Nevertheless, and considering that the respondents could choose more than one option, 223 respondents admitted that they would get a vaccine to observe wild animals, 258 would avoid approaching wild animals while feeling sick, and, importantly, 547 respondents said they would keep their distance from the wild animals. But only 79 respondents would test for COVID-19 and only 162 would wear masks, even though mask usage in Japan was commonplace even before the pandemic outbreak of 2020.

### 3.2 Age-sex class differences

Zoos provide the chance for a great number of tourists from all ages, levels of education, and socioeconomic backgrounds to experience an up-close wildlife encounter, without compromising natural habitats (Doodson et al., 2022) and simultaneously being a source of domestic tourism which is expected to recover faster than international tourism (Toyama, 2022; Kruger and Viljoen, 2023). While we categorized age and sex as variables in our design, we acknowledge that other factors (social context, tradition, demography, etc.) may have impacted visitors' conservation awareness or wildlife connection (Kruger and Viljoen, 2023). Nevertheless, age and sex are important factors to be considered when studying peoples' perceptions and attitudes toward wildlife, as they may influence results to a greater extent than the actual animals' welfare, health, and living environment (Alba et al., 2023). Some previous research has found females and younger age groups to have a more positive perception of wildlife, with women being more concerned about individual welfare and men being more concerned about species conservation (Figueredo et al., 2022; Alba et al., 2023). In addition, younger generations tend to be more critical of captivity in relation to animal welfare (Alba et al., 2023; Gurusamy et al., 2015) particularly in zoos, considering the limitations imposed on animals in exhibiting their natural behavioral repertoire (Pacheco and Madden, 2021) that often lead to increased stress signals and abnormal behavior (Rose et al., 2017) detected by visitors. However, Kruger and Viljoen (2023) found no significant relationship between age and sex in relation to attitudes toward conservation; similarly, a recent meta-analysis by McNally et al. (2024) across 56 studies revealed that age has no significant influence on attitudes toward wildlife. Nevertheless, McNally et al. found that samples with a higher proportion of females reported significantly smaller effect sizes compared to those with fewer females. These findings align with some of our observations, as we did not find marked cross-generational differences. Interestingly, our study also found that men reported higher levels of connection to animals, which contrasts with most findings from Western populations. In this particular case, it may be related to the lack of generational differences in our sampled population. Older Japanese generations may report a connection to the animals according to their religious beliefs, while younger generations may report connection levels in relation to the level of environmental education they have received, regardless of their beliefs. However, we cannot further explore this in our data set, due to the volunteer nature of the recruiting of participants, as male participants or older participants with deeper connections to animals, may have been more likely to accept the questionnaire in the first place, although this may also be true for women and younger participants.

### 3.3 Favorite species

Charismatic species that often drive visitors to zoos and contribute to visitors' satisfaction may be used as models in zoos to attain specific information (e.g., conservation measures for the species and habitat protection) because visitors are more likely to engage in activities related to these species (Colléony et al., 2017; Howell et al., 2019; Consorte-McCrea et al., 2019; but see Mariyam et al., 2022 for similar patterns in nature-based tourism). The endangered gorillas, along with chimpanzees, are one of these charismatic species and a favorite animal of most zoo visitors (Carr, 2016) elsewhere and in our study. Visitors often report feeling easily connected to gorillas and wishing to preserve the species (Myers Jr et al., 2004; Packer et al., 2018). Gorillas‘ flagship status facilitates raising awareness for the conservation of their habitat which also includes other species living within it (Ballantyne et al., 2007). Critically endangered species are thought to have a wider influence on conservation, education, and economy compared to least concern species (Spooner et al., 2023). Aware of such effects, zoos may mediate such species exhibitions to emphasize conservation efforts (Greenwell et al., 2023). For instance, the Maryland Zoo (Unites States) was crucial for the development of the Mountain Gorilla Veterinary Project, operating in the species range countries (Escobar-Ibarra et al., 2021). Other zoos have provided financial support for the development and maintenance of the Mbeli Bai lowland gorilla research field site (e.g., by employing local nationals as rangers or research assistants) and have contributed to local community projects in the same area (Breuer et al., 2018; but Squires et al., 2016; Wilson et al., 2019; Feilen et al., 2018 for examples targeting other species and sites in Africa and South America).

### 3.4 How zoos can promote effective behavioral changes

Visitors often make assumptions about animal welfare based on personal feelings and connections, which may conflict with scientific analysis (e.g., gorillas being naturally inactive at certain ages; Packer et al., 2018). To foster better understanding, zoos must help visitors interpret animal behavior, as familiarity increases care and conservation interest. However, recent studies show visitors are poorly motivated to engage with traditional zoo education programs (Ballantyne et al., 2021). Effective conservation actions should integrate easily into daily routines and offer visible outcomes (Miller et al., 2020). Zoos can highlight direct links between human actions and animal welfare, such as habitat loss due to palm oil production or cobalt mining impacting orangutans and gorillas. They can also address threats from the pet trade (e.g., lemurs, lorises) and entertainment industries (e.g., chimpanzees, macaques). Zoos can suggest actionable steps, from simple to committed, like sponsoring local initiatives, joining community conservation efforts, partnering with wildlife organizations, or raising awareness. By focusing on local issues, zoos can engage visitors in meaningful, region-specific actions to protect animals.

### 3.5 Limitations and future research

It is important to recognize that our survey may not have delivered all the ways that zoo visitors connect with animals and their understanding of conservation and risks associated with close proximity to animals. We aimed to apply a balanced questionnaire, so its length would not impact the visitors' viewing time of the animals. Besides, one of the sampled zoos housed only primate species which may have influenced the data by potentially increasing scores for favorite primate species due to the higher exposure to these animals compared to others. The variation in the layout and design of the informational signage, which is relatively newer and of better quality at HZ, may have influenced the responses. Additionally, JMC offers two interactive exhibits where visitors can enter the enclosures (squirrel monkeys and ring-tailed lemurs island), whereas HZ does not provide this experience, potentially impacting its visitors' sense of connection differently.

We also intended to include a sampled population outside of the zoos for comparison. However, since the Chubu Gakuin sample consists solely of students—most of whom are women and all belonging to Gen Z—its comparison with the more balanced populations from the zoos is also limited. For a better control to the visitor populations, different Nonetheless, this survey is amongst one of the first attempts to study these aspects in Japanese zoos and may serve in the future for a global comparison of zoo visitors' sense of connection with animals. In addition to zoos, future research should also focus on other types of animal housing facilities, such as sanctuaries and recovery centers, as the limited public visiting these locations may have different expectations and learning experiences.

## Data Availability

The original contributions presented in the study are included in the article/Supplementary material, further inquiries can be directed to the corresponding author.
