# Supplementary material for: Reconnection with nature through empathy: rewiring people and animals by assessing zoo visitors' connection to species and the need for their conservation
Source: Front Psychol. 2025 Mar 17;16:1517430. doi: 10.3389/fpsyg.2025.1517430 (PMC11955963; doi:10.3389/fpsyg.2025.1517430)
Supplement: Supplementary file 1 [file Data_Sheet_1.pdf]

Supplementary material Costa et al., submitted

Table 1. The questionnaire sample was applied to participants in the zoo.

Section 1:

Day: ______________ Year of birth: ________________

Hour: _____________ Are you a zoo member? ________

Sex: ______________

Section 2: Connections with Animals at the Zoo

1) What animal did you form the strongest connection with during your visit? (Please write) ___________________________________

2) Please rate the overall connection you felt with the animal.

No connection at all *Some connection* *Strong connection*

1	2	3	4	5	6	7

3) How many times did you visit this animal`s enclosure?
   - ☐ 1
   - ☐ 2-5
   - ☐ 6-10
   - ☐ 10+

4) Approximately how long did you spend at this animal's enclosure in total?
   - ☐ Less than 1 minutes
   - ☐ 1 to 5 minutes
   - ☐ 6 to 10 minutes
   - ☐ 11 to 20 minutes
   - ☐ 21 to 30 minutes
   - ☐ More than 30 minutes

5) To what extent did this animal interact with you (i.e. respond to your movements)? (If this animal shared its enclosure with other individuals, please answer this question about the individual animal you spent the most time watching).
   - ☐ It was asleep
   - ☐ It did not interact with me at all
   - ☐ It interacted with me for less than 5 seconds
   - ☐ It interacted with me for 5 seconds or more

6) Based on your experiences with this animal, please rate your level of agreement with the following items, where 1 = strongly disagree, 4 = neither agree nor disagree, and 7 = strongly agree.

a) My emotional sense of well-being will be severely diminished by the extinction of this species. (Q1)

Strongly disagree		*Neither agree nor disagree*			*Extremely Strongly Agree*	
1	2	3	4	5	6	7

b) I will alter my lifestyle to help protect this species. (Q2)

Strongly disagree		*Neither agree nor disagree*			*Extremely Strongly Agree*	
1	2	3	4	5	6	7

c) My connection to this animal has increased my connection to the species as a whole. (Q3)

Strongly disagree		*Neither agree nor disagree*			*Extremely Strongly Agree*	
1	2	3	4	5	6	7

d) I will learn more about this species in general (e.g., behaviour, natural habitat). (Q4)

Strongly disagree		*Neither agree nor disagree*			*Extremely Strongly Agree*	
1	2	3	4	5	6	7

e) I will learn more about steps I can take to help protect this species. (Q5)

Strongly disagree		*Neither agree nor disagree*			*Extremely Strongly Agree*	
1	2	3	4	5	6	7

f) This animal looked content in its enclosure. (Q6)

Strongly disagree		*Neither agree nor disagree*			*Extremely Strongly Agree*	
1	2	3	4	5	6	7

j) I would purchase an item from the gift shop that supports the conservation of this species. (Q7)

Strongly disagree		*Neither agree nor disagree*			*Extremely Strongly Agree*	
1	2	3	4	5	6	7

Section 3: Existing connection to wildlife

1. Please rate your level of agreement with the following items, where 1 = strongly disagree, 4 = neither agree nor disagree, and 7 = strongly agree.

a) I actively seek opportunities to view wildlife. (Q8)

Strongly disagree		*Neither agree nor disagree*				*Strongly agree*
1	2	3	4	5	6	7

b) I am highly motivated by the need to interact with wildlife. (Q9)

Strongly disagree		*Neither agree nor disagree*				*Strongly agree*
1	2	3	4	5	6	7

c) I spend a lot of time learning about wildlife. (Q10)

Strongly disagree *Neither agree nor disagree* *Extremely Strongly Agree*

1	2	3	4	5	6	7

d) Government officials' views on wildlife are a major factor in my voting decisions. (Q11)

Strongly disagree *Neither agree nor disagree* *Extremely Strongly Agree*

1	2	3	4	5	6	7

e) Even when they are more expensive or harder to find, I will buy groceries and products that support wildlife conservation. (Q12)

Strongly disagree *Neither agree nor disagree* *Extremely Strongly Agree*

1	2	3	4	5	6	7

f) I enjoy watching wildlife. (Q13)

Strongly disagree *Neither agree nor disagree* *Extremely Strongly Agree*

1	2	3	4	5	6	7

j) Wild animals can transmit diseases to humans. (Q14)

Strongly disagree *Neither agree nor disagree* *Extremely Strongly Agree*

1	2	3	4	5	6	7

k) Humans can transmit diseases to wild animals. (Q15)

Strongly disagree *Neither agree nor disagree* *Extremely Strongly Agree*

1	2	3	4	5	6	7

l) Getting close to wild animals is dangerous. (Q16)

Strongly disagree *Neither agree nor disagree* *Strongly agree*

1	2	3	4	5	6	7

m) Getting close to wild animals may lead to:

- ☐ disease
- ☐ injury
- ☐ better picture
- ☐ clearer observation of the animal
- ☐ habituating animals towards humans

n) In order to view wild animals in their habitat, I would (choose one or more options):

- ☐ get vaccinated
- ☐ not going while feeling sick
- ☐ test PCR or antigen for COVID-19
- ☐ use mask
- ☐ pay an extra fee
- ☐ keep the safety distance

Thank you for participating in this survey. Is there anything else you would like to add about your experience at the zoo today? (please write)

(end of the questionnaire)

Table 2. Generation (with the average birth year) and sex distribution of the zoo attendees at Higashiyama and JMC. We classify Gen Z as individuals born between 1995 and 2015; Millennials between 1980 and 1994; Gen X between 1965 and 1979; and Baby Boomers as those born before 1964.

Population	Sex	Generation	No. respondents
Higashiyama	Blank		3
	Female	Baby boomer ($\bar{x}$ birth year 1956)	45
		Gen X (1970)	57
		Gen Z (1999)	24
		Millennial (1986)	42
		Blank	9
	Male	Baby boomer (1954)	39
		Gen X (1971)	26
		Gen Z (1999)	21
		Millennial (1986)	30
		Blank	7
	Other	Gen Z (2001)	1
JMC	Blank	Gen X (1975)	1
		Gen Z (2003)	3
		Millennial (1982)	2
		Blank	4
	Female	Baby boomer (1960)	14
		Gen X (1974)	42
		Gen Z (1999)	36
		Millennial (1985)	55
		Blank	12
	Male	Baby boomer (1957)	14
		Gen X (1972)	32
		Gen Z (1999)	14
		Millennial (1984)	30
		Blank	11

Table 3. A comparison was conducted between the composite variables identified in Howell et al., 2019, and the selected questions utilized in the present study. Cronbach's Alpha was employed to assess internal consistency following the division of composite variables as used in Howell's study. The findings from both studies suggest that "Species-specific conservation caring" demonstrated consistent scoring across the two studies.

**Composite variable name**	**Composite and individual conservation-related items provided by Howell et al., 2019**	**Cronbach 's α**	**Items used in the present study**	**Cronbach's α of selected questions for comparison**	
				Population	Cronbach's α
**Existing connection to wildlife**	I actively seek opportunities to view wildlife	0.8	Q8	JMC,<br>HZ<br>S22B,<br>S22A,<br>S23B,<br>S23A	0.64<br>0.68<br>0.65<br>0.68<br>0.70<br>0.69
	I feel a deep connection to wildlife		x		
	I am highly motivated by the need to interact with wildlife		Q9		
	I spend a lot of time learning about wildlife		Q10		
**Species-specific conservation caring**	My emotional sense of well-being will be severely diminished by the extinction of this species	0.87	Q1	JMC<br>HZ<br>S22B<br>S22A<br>S23B<br>S23A	0.86<br>0.83<br>0.78<br>0.83<br>0.91<br>0.89
	Ensuring this species' survival is my highest priority		x		
	I need to learn everything I can about this species		x		
	I would protest Zoos Victoria if I learned of the mistreatment of this animal		x		
	I will alter my lifestyle to help protect this species		Q2		
	My connection to this animal has increased my connection to the species as a whole		Q3		
	I will learn more about this species in general (e.g., behavior, natural habitat)		Q4		
	I will learn more about steps I can take to help protect this species		Q5		
	I would purchase an item from the Melbourne Zoo gift shop that supports the conservation of this species		Q7		

**Perceived welfare in enclosure**	There is ample space in this animal's enclosure to allow social interaction as well as alone	0.77	x
	time for individuals if they choose		x
	This animal's enclosure design and features provide for a range of species appropriate		x
	behaviors		x
	This animal looked content in its enclosure		Q6
**Single items**	I will endorse public policy that severely restricts future growth and development in order to protect wildlife		x
	Government officials' views on wildlife are a major factor in my voting decisions		Q11
	Even when they are more expensive or harder to find, I will buy groceries and products that support wildlife conservation		Q12
	Wildlife protection must be society's highest priority		x
	I enjoy watching wildlife		Q13
	I feel a sense of calm when I watch wildlife		x
	I prefer to interact with domestic animals (e.g., cats and dogs) than to seek out wildlife viewing opportunities (reverse-scored)		x
	I prefer other activities (e.g., watching movies; dining with friends) to watching wildlife (reverse-scored)		x
	x		Q14 (Wild animals can transmit diseases to humans)
	x		Q15 (Humans can transmit diseases to wild animals)
	x		Q16 (Getting close to wild animals is dangerous)

Table 4. Summary of Bayesian model analysis focusing on the estimated effects of "Low" connection levels on zoo visitors' responses (Q1-Q16). The predictor variable “Connection” was divided into “Low” (Likert items 1, 2 and 3) and “High” (items 5, 6 and 7), removing the "4" (medium) responses from the data to address the issue of potential imbalance on a 7-point Likert scale. This helps avoid skewing the results towards either end of the scale (1, 2, and 3 versus 5, 6, and 7). Priors were specified with 4 chains and 2000 iterations. All tests were conducted in R version 4.3.1. The significance of the effects is assessed by examining whether the 95% credible intervals include zero.

Question	Estimated Effect of "Low" Connection	95% Credible Interval	Significance
**Q1**	-0.917	-1.285 to -0.551	Significant
**Q2**	-0.860	-1.248 to -0.474	Significant
**Q3**	-1.368	-1.773 to -0.974	Significant
**Q4-Q15**	Ranges from small to moderate	Does not contain zero	Significant
**Q16**	0.123	-0.251 to 0.498	Not Significant

Table 5. Sex and age class differences among participants in regards to their connection rates.

Sex	Generation	Mean Rate of Connection	Standard Deviation	Number of Responses
Blank	Blank	3.714285714	1.49602648	7
	Gen X	6	Blank	1
	Gen Z	5	1	3
	Millennial	3.5	0.70710678	2
Female	Baby boomer	3.745454545	1.61266035	55
	Blank	4.047619048	1.65759436	21
	Gen X	3.793814433	1.52026686	97
	Gen Z	3.913793103	1.54788461	58
	Millennial	3.547368421	1.28633093	95
Male	Baby boomer	4.235294118	1.42250814	51
	Blank	4.357142857	1.73680267	14
	Gen X	4.037735849	1.49309003	53
	Gen Z	4.2	1.53009035	35
	Millennial	3.933333333	1.4713843	60
Other	Gen Z	7	Blank	1

Table 6. Influence of Time Spent and Type of Interaction with the animals on Visitors' Connection Rates.

		**Mean Rate of Connection**	**Standard Deviation**	**Number of Responses**
**Time**	Blank	3.430303	1.498879	165
	Long	4.484848	1.581065	66
	Medium	4.154472	1.425945	123
	Short	3.711538	1.466533	364
**Type of interaction**	Blank	3.497326	1.486044	187
	Long	4.643939	1.272928	132
	No	3.330357	1.487698	224
	Short	4.062857	1.402601	175

*Time spent with the animals was categorized between "Short" (less than 10 minutes), "Medium" (between 11 to 30 minutes) and "High" (more than 30 minutes); and Interaction made with the animals were categorized between "No" (absent), "Short" (less than 5 seconds) or "Long" (more than 5 seconds).
